# Vapor pressure deficit control and mechanical vibration techniques to induce self-pollination in strawberry flowers

**DOI:** 10.1186/s13007-025-01343-2

**Published:** 2025-02-25

**Authors:** Hyein Lee, Meiyan Cui, Byungkwan Lee, Jeesang Myung, Jaewook Shin, Changhoo Chun

**Affiliations:** 1 Department of Agriculture, Forestry and Bioresources, Seoul National University, Seoul, 08826 Korea; 2 Research Institute of Agriculture and Life Sciences, Seoul National University, Seoul, 08826 Korea

**Keywords:** Pollination strategy, Self-pollination, Strawberry flower, Vibration pollination, VPD conditions

## Abstract

**Background:**

Pollination strategies to supplement or replace insect pollinators are needed to produce marketable strawberry fruits in indoor vertical farms. To ensure the self-pollination of strawberry flowers, anther dehiscence, and pollen attachment were investigated under different vapor pressure deficit (VPD) conditions and external mechanical wave vibrations.

**Results:**

The proportion of dehisced anthers was examined under VPDs of 2.06, 1.58, and 0.33 kPa, and the projected area of pollen clumps was assessed under VPDs of 2.06 and 0.33 kPa. After exposing flowers to a VPD of 2.06 kPa, vibrations with various frequency (Hz) and root mean square acceleration (m s^−2^) combinations were used to evaluate pollination effectiveness. The anthers underwent complete dehiscence at VPDs of 2.06, 1.58, and 0.33 kPa. The pollen clump ejection index was highest at a VPD of 2.06 kPa. Pollen clump detachment was effective at 800 Hz with 40 m s^−2^, while pollen attachment to the stigma was most effective at 100 Hz with 30 and 40 m s^−2^.

**Conclusions:**

These findings demonstrate that high VPD promotes anther dehiscence timing and facilitates pollen clump formation, while specific vibration frequencies with high acceleration optimize pollen detachment and stigma attachment, offering an effective strategy for controlled strawberry pollination in vertical farming.

## Background

Strawberry (*Fragaria* × *ananassa* Duch.) cultivation has been attempted in indoor vertical farming systems [1–3], which allow for maximizing yield per unit area through multilayer cultivation. The active control of environmental factors in vertical farms enables cultivation regardless of the region and climate [4]. This further allows for potential year-round production of high-quality fruits, and the pollination of strawberry flowers is crucial for the success of indoor vertical farming. In indoor vertical farming systems, hand or insect pollination is used to produce marketable fruits. However, hand pollination is labor-intensive [5, 6] and insect pollination can still lead to the production of malformed fruits [7, 8]. The activity of pollinators is highly influenced by environmental conditions, with reduced activity under high relative humidity [9], and in limited spaces, malformed fruit can occur when the number of bees exceeds the number of plants, as frequent visits reduce stigma receptivity [10]. The difficulty in ensuring pollinator activity in limited indoor spaces combined with environments unsuitable for pollinators can further increase the production of malformed fruits. Therefore, strategies for strawberry flower pollination are required to supplement or replace insect pollination in indoor vertical farming. Examining the strawberry flower structure and pollination characteristics is essential for developing such strategies.

A typical strawberry inflorescence develops from a fruit bud, with the peduncle and pedicels extending from the stem, and consists of a primary flower on the main axis, branching into two secondary flowers, four tertiary flowers, and eight quaternary flowers [11]. Strawberry flowers are hermaphrodites and possess the morphological elements necessary for self-pollination; however, self-pollination is often insufficient to pollinate the whole flower. The stamens of strawberry flowers are arranged in a particular pattern, with 5 tall and 5 short stamens each in the inner whorl, 10 medium-length stamens in the outer whorl, and the central receptacle topped with numerous spirally arranged carpels [12]. When the pistils in the flowers are evenly pollinated and more than 70–80% of the pistils are fertilized, the flower can develop a normal fruit shape [13]. However, incomplete pollination usually results in poor or malformed fruit sets. Thus, the majority of the hundreds of pistils need to be pollinated to produce marketable strawberries [8].

Self-pollination occurs when the strawberry pollen matures and is released through lateral slits in the anthers. The pollen is occasionally released under tension and attached to the pistils, which may be in close contact with the anthers [14]. However, owing to the unique structure of strawberry flowers, achieving successful self-pollination without additional aids is challenging. Alternative pollination tools, such as an ultrasonic pollinator [15] and a pollination robot [16], have been used to improve self-pollination in strawberries. These attempts demonstrate the potential of mechanical pollination methods. However, existing pollination methods often produce malformed strawberries and are yet to replace insect pollination.

In many species, anthers remain hydraulically connected to the flower until dehiscence, sometimes preceded by rapid filament extension [17–19], with both dehiscence and pollen release, preceding conditions of pollination, significantly influenced by microclimatic factors such as air temperature and relative humidity, which can be explained as vapor pressure deficits (VPDs). In nature, the proportion of dehisced anthers is the highest during midday, coinciding with peak pollinator activity [20]. Consequently, increasing temperature and decreasing relative humidity enhance the proportion of dehisced anthers [21–24]. These characteristics can be strategically utilized to develop pollination methods for indoor vertical farming systems where environmental control is more feasible.

Plants pollinated via insect pollinators do not release pollen all at once but gradually, and the vibration induced by insect pollinators can increase pollen release [25]. Considering that bees use their indirect flight muscles in the thorax to release pollen from anthers during pollination [26–29], it is possible to induce pollen release and pollination through external mechanical vibrations as an alternative to pollination via bees. Theoretically, the vibration of flowers at the natural frequency could result in greater pollen release owing to the increased acceleration associated with resonance [30]. However, the properties of anthers, such as their shape, length, and slit size, can affect pollen release [27], thus making it necessary to identify the vibration characteristics suitable for strawberry pollination.

Experiments were conducted to effectively induce pollen release through environmental control before applying of mechanical vibrations for pollination. Mechanical vibrations with high practical potential were used as a pollination method to improve the success rate of self-pollination in an indoor vertical farming system. Additionally, the study aimed to identify the vibration characteristics that are effective for strawberry pollination. This study investigated the possibility and efficiency of mechanical pollination systems in indoor vertical farming systems.

## Materials and methods

### Plant materials and environmental conditions in a plant factory

**Fig. 1:**
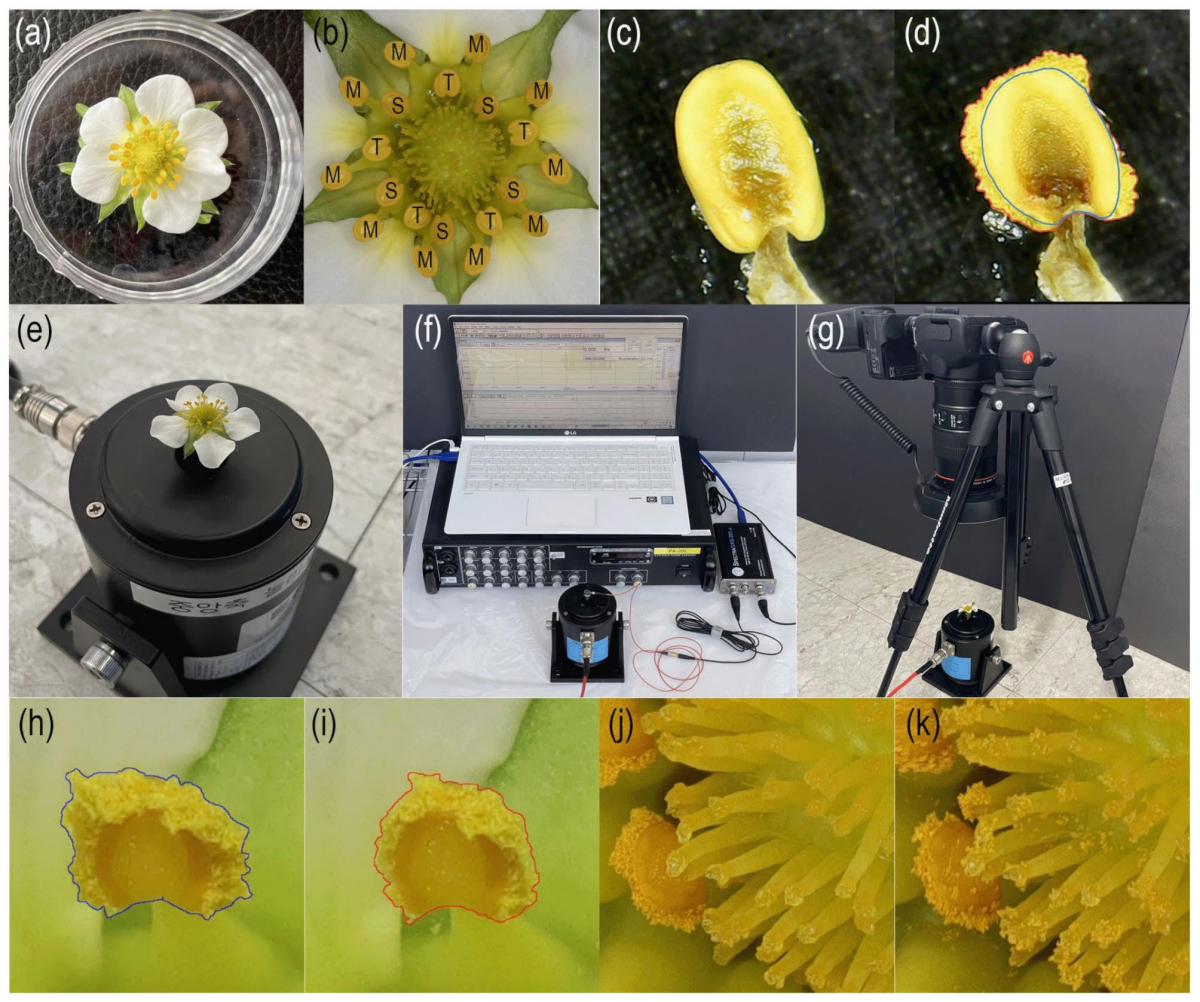
Images of experimental materials, equipment, and methods. Sampled strawberry flower before exposure to VPD conditions (**a**), stamen types of strawberry flower: short (S), medium (M), and tall (T) (**b**), before anther dehiscence (**c**), the projected area of anther (blue line) and pollen clump (red line) after anther dehiscence (**d**), flower affixed onto the axis of the shaker (**e**), vibration equipment (**f**), camera for photographs before and after vibration treatments (**g**), the projected area of anther and pollen clump (blue line) before vibration treatment (**h**), the projected area of anther and pollen clump (red line) after vibration treatment (**i**), flower pistils before vibration treatment (**j**), and flower pistils after vibration treatment (**k**)

Strawberry transplants (*Fragaria* × *ananassa* Duch. ‘Seolhyang’) were produced via an autotrophic transplant production method in a plant factory with artificial lighting [31, 32]. On July 28, 2023, 72 transplants were transplanted and cultivated in a plant factory using white LEDs (Future Green Co., Ltd., Yongin, Korea) with a photosynthetic photon flux density of 200 μmol m^−2^ s^−1^ and a photoperiod of 12 h d^−1^. Air temperature and relative humidity were maintained at 23 °C /12°C and 60%/90% (during photo-/dark periods), respectively. The CO_2_ concentration was 500 μmol mol^−1^, and the strawberry plants were fertigated with the Yamazaki nutrient solution for strawberries (N, 5.5 me L^−1^; P, 1.5 me L^−1^; K, 3.0 me L^−1^; Ca, 2.0 me L^−1^; Mg, 1.0 me L^−1^; S, 1.0 me L^−1^) at pH 6.5 and EC 1.5 dS m^−1^ using a drip fertigation system. A total of 138 fully opened flowers before anther dehiscence were collected from the plants for the experiments (Fig. 1a). The stamens of the strawberry flowers were classified into three types, as described by Hollender [12], and the results were categorized according to the stamen types. Tall and short-length stamens were arranged in a repeating pattern in the inner whorl, and medium-length stamens were arranged in the outer whorl (Fig. 1b).

### VPD treatments

**Fig. 2:**
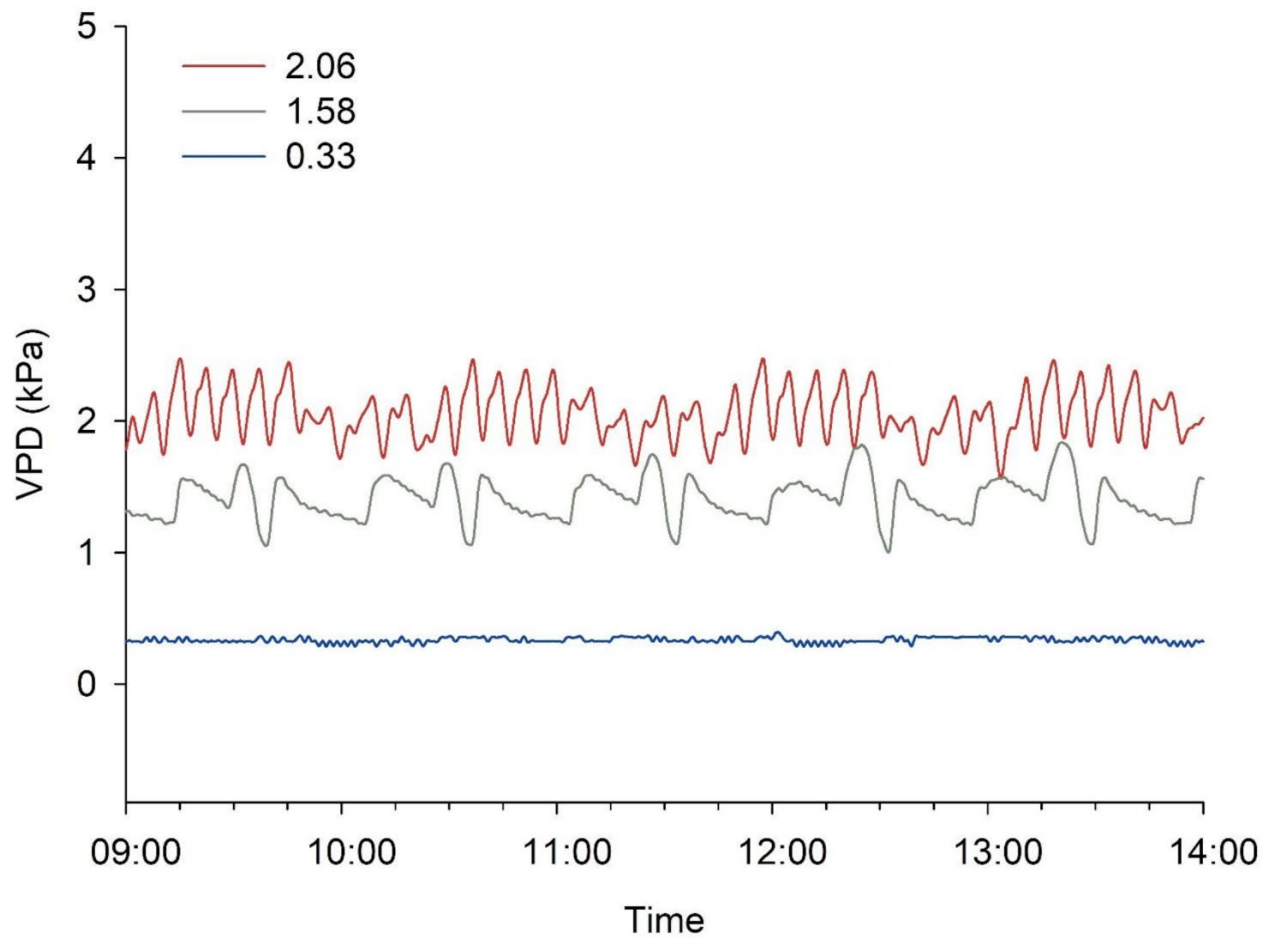
Vapor pressure deficit (VPD) level in a growth chamber set at 25 °C. Relative humidity levels of 35%, 50%, and 90% corresponded to VPD values of 2.06, 1.58, and 0.33 kPa, respectively

The temperature of the growth chamber was 25 °C with a relative humidity of 35%, 50%, and 90% (VPD conditions of 2.06, 1.58, and 0.33 kPa, respectively; Fig. 2). The flowers were moved to the growth chamber for exposure to each VPD condition.

### VPD effects on anther dehiscence

The anthers were observed using a portable microscope SMTUSCOPE (Shenzhen Qi Yao Technology Co., Ltd, Shenzhen, China) installed within the chamber. Images were captured to determine the anther dehiscence and quantify the ejected pollen clumps (Fig. 1c, d). The results were then classified according to the type of stamen.

Flowers were placed in growth chambers with VPD conditions of 2.06, 1.58, and 0.33 kPa, respectively, and observed for 300 min at 10 min intervals to determine the timing of anther dehiscence. This determination was made by counting the anthers that began releasing pollen, and the experiment was conducted in triplicates with three flowers each. The percentage of anther dehiscence was estimated using the following equation:

1 $$\begin{array}{l}\:Percentage\:of\:anther\:dehiscence = \\\frac{{The\:EquationNumber\:of\:dehisced\:anthers}}{{Total\:EquationNumber\:of\:anthers}}\left( \% \right)\end{array}$$

The flowers were placed in growth chambers with VPD conditions of 2.06 and 0.33 kPa and observed for 6 h at hourly intervals to measure the projected area of pollen clumps. Following anther dehiscence, the ejected pollen clumps were observed to quantify the projected area of the pollen clumps, and the experiment was conducted with three flowers. The pollen clump ejection index was calculated using the following equation:

2 $$\begin{array}{l}\:Pollen\:clump\:ejection\:index = \\\frac{{Projected\:area\:of\:anther + Projected\:area\:of\:pollen\:clumps}}{{Projected\:area\:of\:anther}}\end{array}$$

The freehand selection tool in the Fiji software package (http://fiji.sc/Fiji) was used to delineate and draw contours for the visually segmented parts. This approach was utilized to partition the projected area of anther and pollen clumps, serving as the target subjects. Pollen clumps were considered ejected from the anther when the pollen clump ejection index value was > 1.

### Vibration treatments

The experimental setup is shown in Fig. 1e, f, and g. Vertical sine wave vibration was applied using a Modal Shaker MS-20 (YMC Piezotronics Inc., Yangzhou, China), which was connected to a signal amplifier for controlling the accelerations and the sound card Spectra DAQ-200 (Pioneer Hill Software LLC, Sequim, WA, USA). The wave signals were set up using Spectra PLUS spectrum software (Pioneer Hill Software LLC, Sequim, WA, USA) and generated digitally. The accelerometer DYTRAN3225F6 (Dytran Instruments Inc., Chatsworth, CA, USA) was mounted on a round head screw secured within the axis of the shaker to measure frequency and root mean square accelerations (G_rms_) before the experiment. The accelerometer was connected to the sound card Spectra DAQ-200, and the analysis software Spectra PLUS spectrum was used to analyze received signals.

Flowers collected from the plant were affixed directly onto a round head screw using instant adhesive and were placed in the growth chamber at a VPD condition of 2.06 kPa. After 3 h, the flowers were moved to a vibration equipment and secured within the axis of the shaker to apply different combinations of frequencies (100, 200, 400, 800, and 1600 Hz) and G_rms_ (20, 30, and 40 m s^−2^) of vertical sine wave vibration treatments. The vibration was applied for 20 s, followed by a 10 s rest, and repeated thrice.

### Vibration effects on pollination

The focus stacking technique was used to evaluate the effects of vibration treatments. The anthers and pistils of the flowers were photographed using the digital camera EOS 200D II (Canon Inc., Tokyo, Japan) equipped with the macro lens Canon EF 100 mm f/2.8 L Macro IS USM (Canon Inc., Tokyo, Japan) (Fig. 1g). Macrophotography was performed under ISO 200, F/8.0, and 1/15 conditions to analyze the overall changes rather than focusing on specific parts. The strawberry flowers were photographed at different focal depths to apply the focus stacking technique. The conditions for macrophotography for the focus stacking technique were adapted from the description provided by MacInnis and Forrest [33].

A combination of frequencies and G_rms_ of vertical sine wave vibrations was applied. Images of the flowers were captured before and after vibration treatments to evaluate the pollen clump detachment under the vibration treatments (Fig. 1h, i), and the experiment was conducted using three flowers. The percentage of detached pollen clumps following vertical vibration treatments was calculated using the following equation:

3 $$\begin{array}{l}\:Detached\:pollen\:clumps\:after\:vertical\:vibration = \\\frac{{Projected\:area\:of\:pollen\:clump\:before\:vibration - after\:vibration}}{{Projected\:area\:of\:pollen\:clump\:before\:vibration}}\:\left( \% \right)\end{array}$$

Following the vibration treatments, the images of the flowers were analyzed to evaluate the pollination efficiency under the vibration treatments (Fig. 1j, k). The experiment was conducted using the same three flowers. The percentage of stigma with attached pollen after the vibration treatments was estimated using the following equation:

4 $$\begin{array}{l}\:Stigma\:with\:attached\:pollens\:after\:vibration = \\\frac{{The\:EquationNumber\:of\:pollinated\:stigma}}{{Total\:EquationNumber\:of\:pistil}}\:\left( \% \right)\end{array}$$

The freehand selection and cell counter tools in the Fiji program were used to visually delineate and draw contours for segmentation and count the number of pistils, respectively. This approach was utilized to partition the projected area of pollen clumps before and after vibration, serving as the target subjects, and to count the total number of pistils and pollinated stigma.

### Paraffin sections

The anthers were fixed in an FAA solution (10% [v/v] formaldehyde, 5% [v/v] acetic acid, and 50% [v/v] ethanol) overnight. The anthers were subsequently dehydrated in each ethanol gradient (twice at 50%, 70%, 80%, 90%, and 100%) for 10 min each, followed by 100% ethanol for 60 min. The anthers were cleared in xylene/ethanol solutions of increasing concentration (25%, 50%, and 75%) for 60 min each and then twice with 100% xylene for 90 min each. Then, the anthers were incubated in a 50% xylene/molten paraffin wax solution at 65 °C overnight and then incubated twice in 100% molten paraffin wax at 65 °C overnight before being embedded in paraffin wax. The anthers were embedded in paraffin wax by placing the sample into the embedding frame before the wax solidified and melted wax was poured into the embedding frame. To observe the anthers, the embedded blocks were sectioned at 10 μm and stained with 0.1% toluidine blue.

### Statistical analysis

The data were analyzed using SAS software version 9.4 (SAS Institute Inc., Cary, NC, USA). For parameters showing significant results in one-way ANOVA, with *P* < 0.05 considered statistically significant, Tukey’s HSD test was used for multiple comparisons, and Student’s t-test was applied for pairwise comparisons. Two-way ANOVA was performed to determine significant interaction effects, with *P* < 0.05 considered statistically significant. The data were visualized using SigmaPlot 10.0 (Systat Software, Inc., San Jose, CA, USA).

## Results

### Effect of VPD conditions on anther dehiscence time

**Fig. 3:**
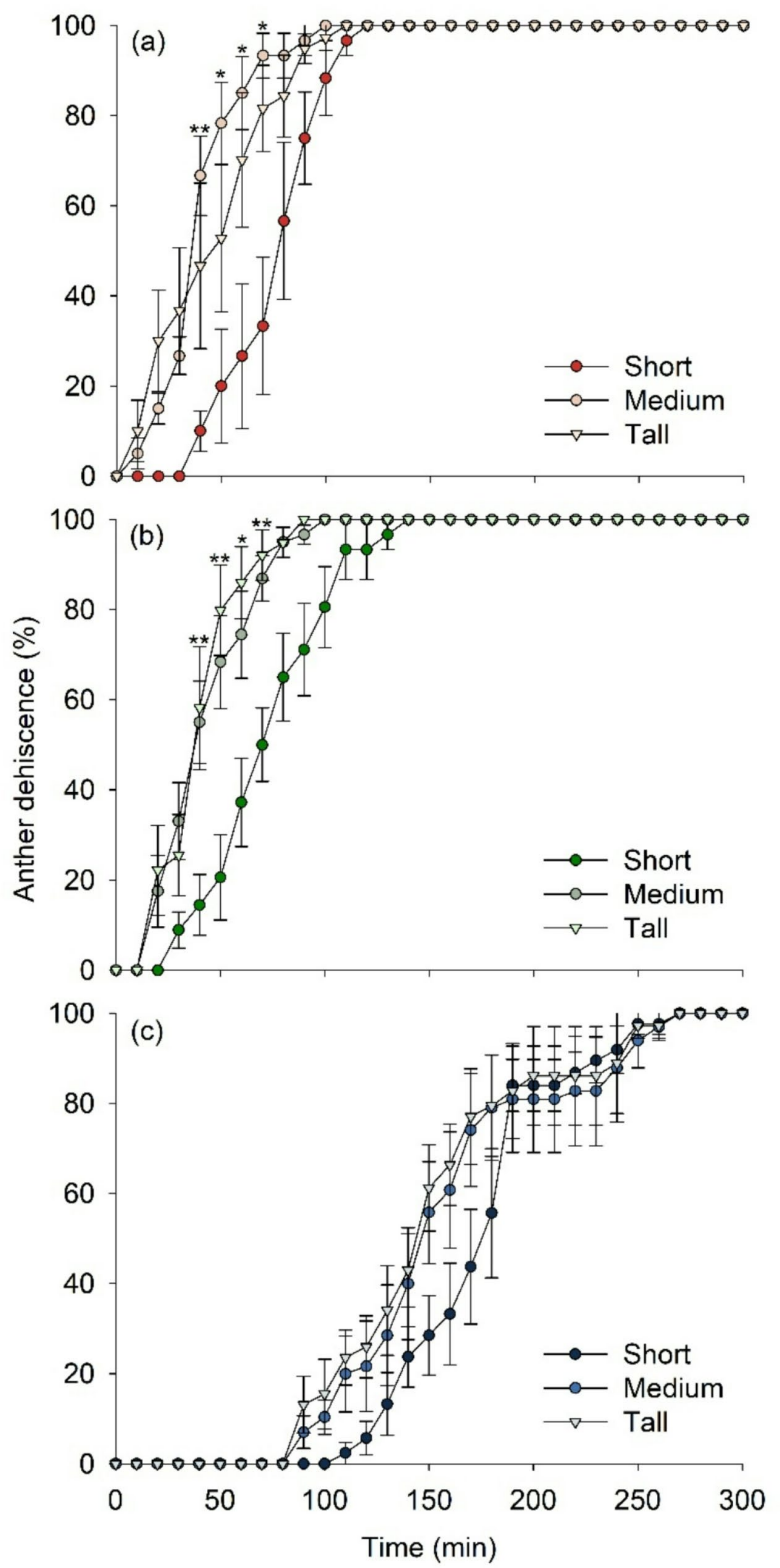
Percentage of the anther dehiscence of strawberry anthers. Short, medium, and tall stamens under vapor pressure deficit of 2.06 (**a**), 1.58 (**b**), and 0.33 (**c**) kPa. Asterisks indicate significant differences between the short, medium, and tall stamen types at each time point, as determined by Tukey’s HSD test following a significant ANOVA result, at *P* = 0.05 (*) or 0.01 (**)

The timing of anther dehiscence varied depending on the VPD conditions and the types of stamens. The earliest anther dehiscence was observed under a VPD condition of 2.06 kPa (Fig. 3a), whereas a VPD condition of 0.33 kPa delayed anther dehiscence (Fig. 3c). Anthers in flowers fully dehisced at 120, 140, and 270 min under VPD conditions of 2.06, 1.58, and 0.33 kPa, respectively (Fig. 3). In the 2.06 kPa VPD condition, anther dehiscence began at 10 min for the medium and tall types of stamens with significant differences observed between the stamen types at 40–70 min (Fig. 3a). The order of complete anther dehiscence was medium, tall, and short stamens. In the 1.58 kPa VPD condition, anther dehiscence began at 20 min for the tall and medium types of stamens (Fig. 3b), with a similar order of complete anther dehiscence as observed in the 2.06 kPa VPD condition (Fig. 3a). Anther dehiscence was delayed in the short type of stamen compared with that under the 2.06 kPa VPD condition, with significant differences observed between the stamen types at 40–70 min (Fig. 3b). In the 0.33 kPa VPD condition, anther dehiscence began at 90 min for the tall and medium stamen types and at 110 min for the short stamen type (Fig. 3c).

### Pollen release affected by the VPD conditions

**Fig. 4:**
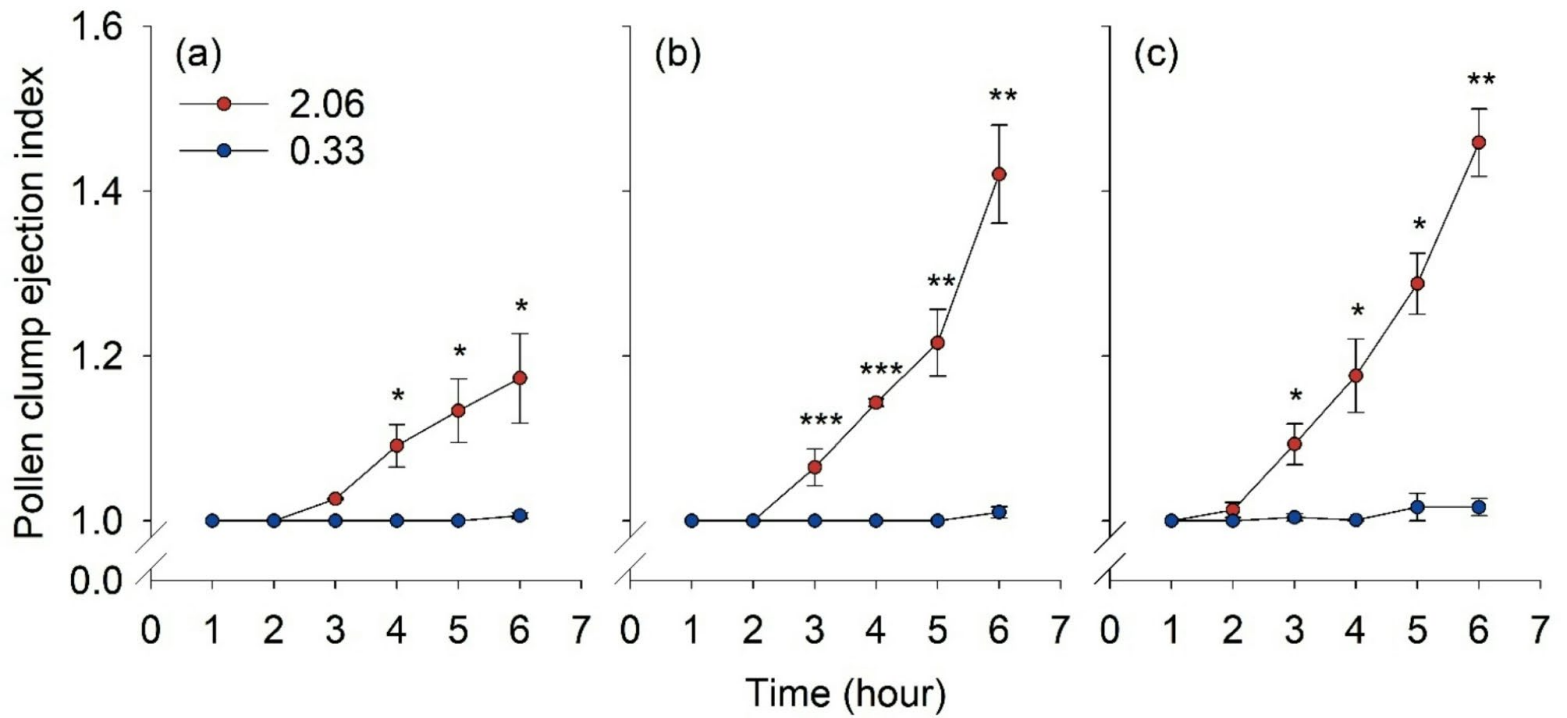
Pollen clumps ejection index of strawberry anthers. Short (**a**), medium (**b**), and tall (**c**) stamens under vapor pressure deficit of 2.06 and 0.33 kPa. Asterisks indicate significant differences between 2.06 and 0.33 kPa treatments at each time point via Student’s *t*-test at *P* = 0.05 (*), 0.01 (**), or 0.001 (***)

**Fig. 5:**
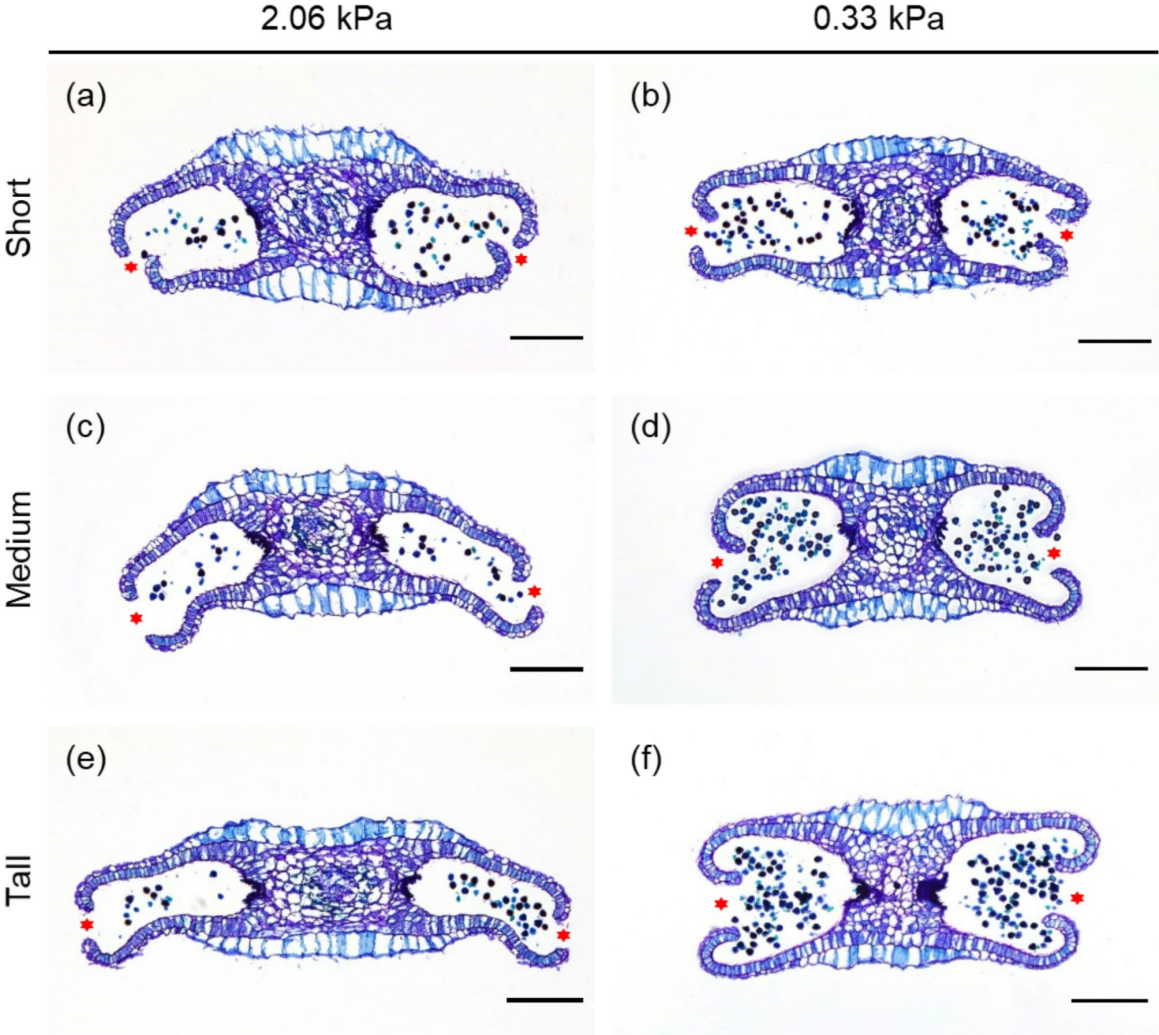
Transverse section of strawberry anthers after exposure to a vapor pressure deficit for 3 h. vapor pressure deficit level of 2.06 kPa (**a**, **c**, **e**) and 0.33 kPa (**b**, **d**, **f**). The opened area is marked with a red hexagram. Bars = 200 μm

The pollen clump ejection index varied significantly depending on the VPD conditions and the types of stamens. At 3 and 6 h, the pollen clump ejection index of the short and medium stamens increased to more than 1 under VPD conditions of 2.06 and 0.33 kPa, respectively (Fig. 4a, b). For tall stamens, the index increased to more than 1 at 2 h under VPD conditions of 2.06 and 0.33 kPa (Fig. 4c). Significant differences between the two VPD conditions were observed for the medium and tall stamens from 3 h and for short stamen from 4 h (Fig. 4). The short stamen type demonstrates a lower value compared to the medium and tall stamens, under a VPD condition of 2.06 kPa. The anthers dehisced after 3 h exposure to a VPD condition of 2.06 and 0.33 kPa (Fig. 5). A smaller amount of pollen grains remained in the locule at a VPD condition of 2.06 kPa (Fig. 5a, c, e) compared with that at a VPD condition of 0.33 kPa (Fig. 5b, d, f) in all the stamen types. Anther senescence started, and cell structure shrinkage occurred following anther dehiscence at a VPD condition of 2.06 kPa for the medium and tall stamens (Fig. 5c, e).

### Vibration conditions affect pollen detachment from the anther

**Fig. 6:**
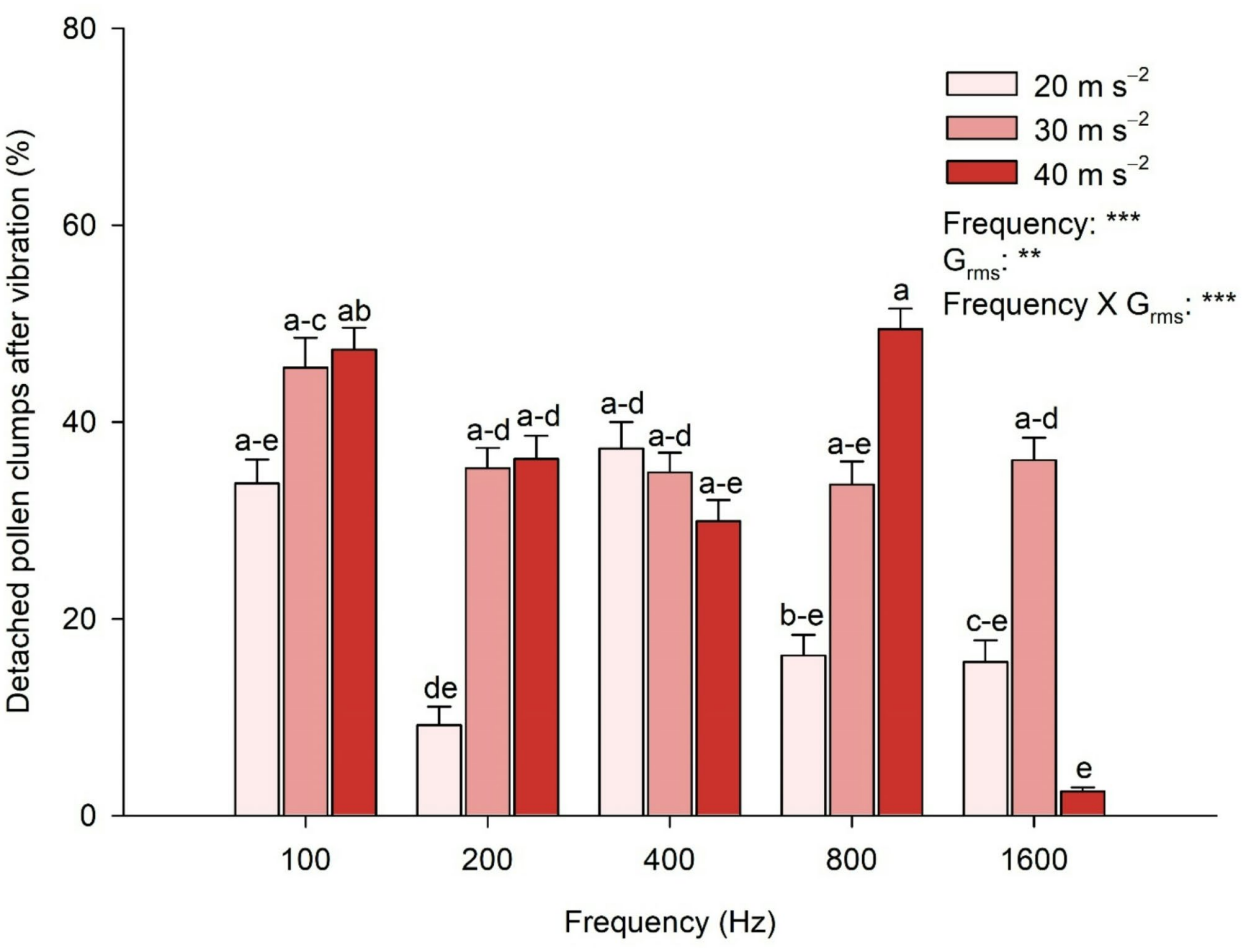
Effect of the vibration treatment on pollen detachment from the anther. The vibration treatments with various frequencies and root mean square accelerations (G_rms_) were employed on the individual flowers after exposure to a vapor pressure deficit of 2.06 kPa for 3 h. Means with different letters above columns are significantly different between G_rms_ of 20, 30, and 40 m s^−2^, according to Tukey’s HSD test at *P* = 0.05. Significant effects of Frequency, G_rms_, and their interaction (Frequency × G_rms_) were determined by two-way ANOVA at *P* = 0.01 (**) or 0.001 (***)

As the vibration treatments detached more pollen clumps, the percentage of the detached pollen clumps increased (Fig. 6). The two-way ANOVA analysis indicated that detached pollen clumps after vibration were significantly affected by frequency and G_rms_ and their interaction (frequency × G_rms_). The highest percentage of detached pollen clumps after vibration was observed at 800 Hz frequencies with 40 m s^−2^ G_rms_. By contrast, the lowest percentage was recorded at 1600 Hz with 40 m s^−2^. Pollen dispersal was more effective with increased G_rms_ across most frequencies, excluding 400 and 1600 Hz. The order of the detached pollen clumps percentage varied across different G_rms_ levels and frequencies, with no significant trend observed between the frequencies.

### Vibration conditions affect pollen attachment to the stigma

**Fig. 7:**
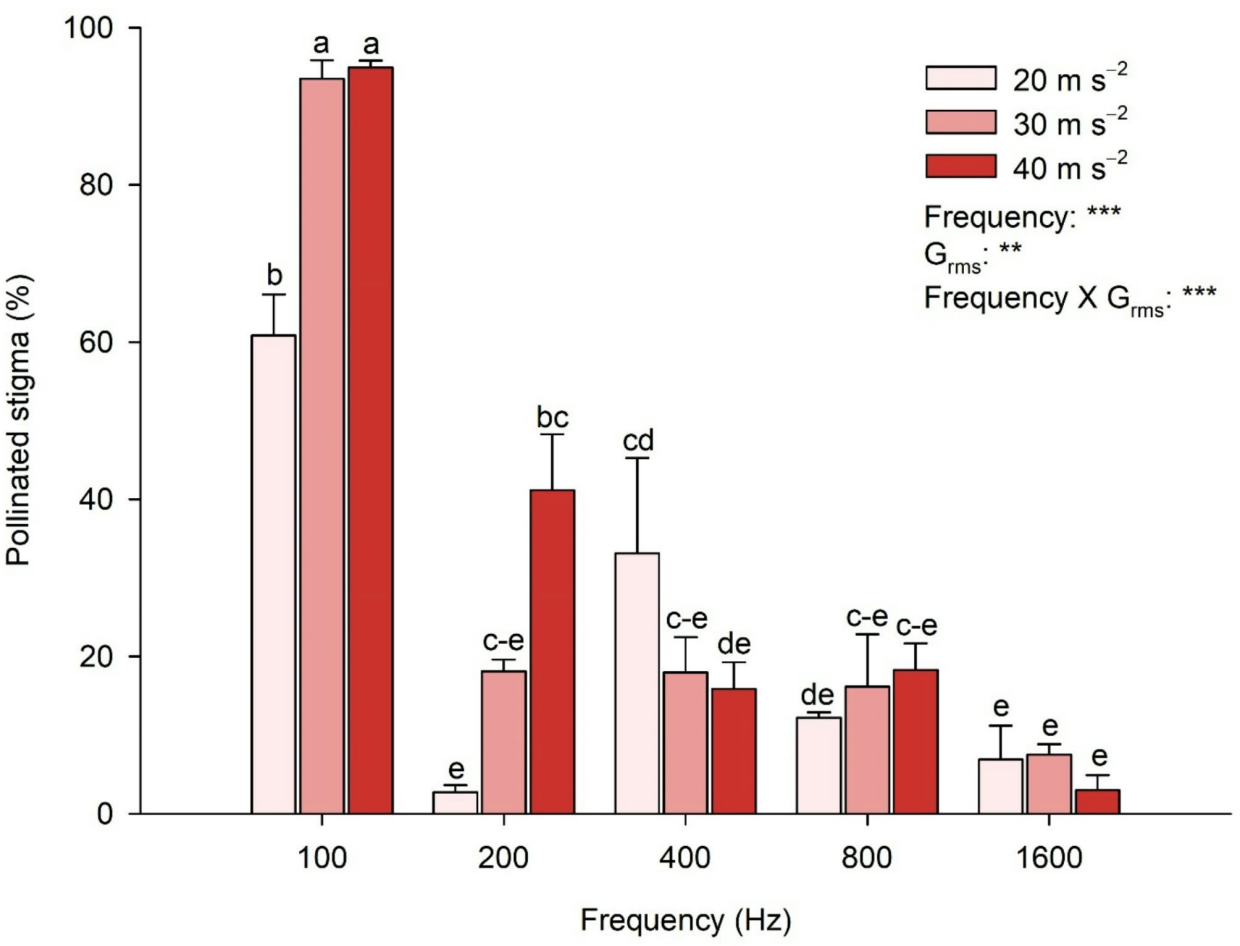
Effect of the vibration treatment on pollen attachment to the stigma. The vibration treatments with various frequencies and root mean square accelerations (G_rms_) were employed on the individual flowers after exposure to a vapor pressure deficit of 2.06 kPa for 3 h. Means with different letters above columns are significantly different between G_rms_ of 20, 30, and 40 m s^−2^, according to Tukey’s HSD test at *P* = 0.05. Significant effects of Frequency, G_rms_, and their interaction (Frequency × G_rms_) were determined by two-way ANOVA at *P* = 0.01 (**) or 0.001 (***)

The pollinated stigmas were distinguished and analyzed following the vibration treatments (Fig. 7). The two-way ANOVA analysis indicated that pollinated stigma after vibration was significantly affected by frequency and G_rms_ and their interaction (frequency × G_rms_). The percentage of pollinated stigmas increased with G_rms_ at 100 and 200 Hz frequencies. The highest percentage of pollinated stigmas was observed at 100 Hz with 30 and 40 m s^−2^, exceeding 90%. This was followed by that at 100 Hz at 20 m s^−2^, which resulted in a percentage of 60%. Treatments at 200 Hz with 40 m s^−2^ and 400 Hz with 20 m s^−2^ resulted in 30–40%.

## Discussion

### Anther dehiscence under VPD conditions

Anther dehiscence, which is the final stage of flower development, involves complex cellular degeneration to allow pollen release. As shown in the paraffin sections of anthers exposed to each VPD condition for 3 h in Fig. 3, anther dehiscence occurs due to cellular degeneration and the shrinkage of the tangential outer wall, ultimately leading to pollen release. Internal water status plays a crucial role in influencing the dehydration of anthers and the shrinkage of the tangential outer wall, thus providing the necessary force for anther dehiscence [34]. Externally, low relative humidity accelerates anther dehiscence, whereas increased relative humidity delays or inhibits this process [21, 35, 36].

According to the tested VPD conditions, relative humidity significantly impacted the timing of strawberry anther dehiscence. Under the same temperature condition, anther dehisced faster at a high VPD condition of 2.06 kPa, where relative humidity was lower. Lower relative humidity promoted dehiscence, and high relative humidity at a VPD condition of 0.33 kPa effectively delayed anther dehiscence (Fig. 3). Similarly, anther dehiscence was delayed with increasing relative humidity, effectively at 97%, whereas the dehiscence of detached anthers accelerated at 33% and 64% in apricot and peach, respectively [22]. In strawberries, anther dehiscence was delayed but still occurred at high relative humidity (Fig. 3c). This indicates that anther dehiscence occurs at a specific developmental stage, regardless of relative humidity. Once the anther dehiscence stage is reached, environmental conditions do not affect the process.

However, this mechanism varies among species, as increased relative humidity inhibited anther dehiscence in *Ricinus communis* L [21]., whereas the species with stomata on the anthers may be more effectively affected by relative humidity conditions [34]. The anthers with the tall type of stamen started to dehisce first and those with the short type of stamen dehisced last in all the VPD conditions (Fig. 3). In *Allium triquetrum*, the anther dehiscence time differed between the inner and outer whorl, which had varying filament lengths according to their positions, with the inner-whorl anthers dehiscing earlier than the outer-whorl anthers [23]. These results indicate that VPD can effectively control the timing of strawberry anther dehiscence, suggesting the possibility of regulation through environmental control before applying mechanical pollination methods.

The process of pollen release after anther dehiscence is also effectively influenced by the VPD conditions. Strawberry pollen releases a clump structure, which is advantageous for self-pollination, while smaller clumps or individual pollen grains are effective for distance cross-pollination [37]. Strawberry pollen has a tectum on the sexine [38] and appears to be released in clumps, influenced by the distribution of pollenkitt on the tectum. Strawberries, suitable for insect and wind pollination, maintain a balance in terms of stickiness [39]. The pollen clump ejection index was determined to evaluate the extent of pollen clump release according to VPD. The anthers were dehisced for 60 min under a VPD condition of 2.06 kPa (Fig. 3a). However, the pollen clump ejection index did not increase at 1 h (Fig. 4) and began to change at 2 h in the tall type of stamen (Fig. 4c). At 3 h, anthers dehisced under all conditions (Fig. 5); however, the pollen clump ejection index varied depending on the VPD conditions and stamen type (Fig. 4). These results indicate that a sufficient amount of time is required for pollen to be released outside after anther dehiscence and high relative humidity conditions inhibit the subsequent release of pollen from the anther.

Cell degeneration and shrinkage were observed in the anthers of the tall and medium types of stamens at 3 h under a VPD condition of 2.06 kPa (Fig. 5). The anthers of the tall and medium stamens were more affected by relative humidity than those of the short stamens. The cause of asynchrony of anther dehiscence in *A*. *triquetrum* was potentially attributed to the timing of anther dehiscence, which might be regulated internally for each anther and whorl [23]. In *Fragaria vesca*, strawberry flower developmental stages 5–7 include stamen initiation and development; at stage 9, medium- and tall-type stamens elongate, and later, at stage 11, filaments of short stamens elongate [12]. The earlier dehiscence of medium and tall stamens and their response to VPD are likely due to the differences in their relative developmental stages. In strawberry flowers, the different dehiscence patterns of stamens of varying heights may be a factor influencing fruit development. When applying mechanical pollination to fertilize the hundreds of pistils spirally arranged on a receptacle, this can be important.

### Mechanical vibration to induce self-pollination

Commercially cultivated strawberries are pollinated using pollinators such as honeybees or bumblebees. Bees generate vibration in various behavioral contexts, including pollen collection [40], suggesting that pollen release is related to the mechanical shaking of anthers caused by the direct physical contact of floral structures with bees [27]. The frequency of floral buzz produced by bees varies depending on the availability of pollen from poricidal flowers [27, 41, 42], with the frequency range measured between 200 and 500 Hz [28, 42, 43]. Various external mechanical shaking methods were applied to disaggregate pollen clumps into smaller clumps or pollen grains through combinations of frequency and G_rms_ vibration. The percentage of detached pollen clumps increased with increasing G_rms_ at most frequencies (Fig. 6). These findings are similar to the suggestion that pollen release is influenced more by acceleration, displacement, or velocity than by the optimal frequency of buzz pollination [44]. In addition, these results align with findings that pollen release depends on the velocity or acceleration of the vibration applied to anthers by bees [27, 45].

Frequencies of 100 and 800 Hz with 40 m s^−2^ more effectively detached pollen than the other frequency and G_rms_ combinations (Fig. 6). These results indicate that specific frequencies are effective for disaggregating the pollen clumps of strawberry flowers. Bumblebees can change the frequency and duration of vibration on numerous flowers to increase the pollen release from the anthers [46, 47]. Additionally, vibrations at the resonance frequency of stamens can increase pollen removal for smaller bees that cannot reach the required acceleration to release pollen [48, 49]. These experimental results indicate that pollen release can be influenced by frequency.

The attached pollen on the stigma was recognized after the vertical vibration treatments. Strawberry flowers are hypogynous, with each pistil forming an individual unit on the surface of a raised receptacle [12]. Marketable fruits do not have deformities owing to the successful pollination of the numerous pistils on the receptacle, which are evenly fertilized by pollen. Deformed strawberry fruit production can be caused by various factors [50], especially damage to flower organs due to extreme environmental conditions during the flower developmental stages [51–54] or insufficient pollination by bees [55–57]. Ensuring sufficient pollination on indoor vertical farms is essential, as these farms provide an optimal cultivation environment throughout the period.

To induce self-pollination through vibration pollination and evaluate its effectiveness, assessing the pollens detached owing to the vibration treatment that pollinated all the flower pistils is necessary. As a result of the detached pollen clump, 100 and 800 Hz frequencies were effective. However, the detached pollens were most evenly distributed on the pistils at 100 Hz (Figs. 6 and 7). Notably, a frequency of 100 Hz was relatively effective even at the lowest G_rms_, with over 90% at 30 and 40 m s^−2^ (Fig. 7). The effects of vibration vary depending on the floral morphology, as the arrangement of stamens, with anthers either tightly held together or loosely arranged, influences vibration transmission [58]. The anthers of strawberry flowers are loosely arranged, and such floral structures would benefit from whole-flower vibration for effective pollination. To develop effective mechanical pollination systems, pollens detached from clumps must adhere evenly to the stigma during the pollination process. Considering all factors, the most effective vibration frequency for pollinating strawberry flowers was 100 Hz.

### Development of mechanical pollination system

Developing a novel pollination system is essential in indoor vertical farming because of several challenges associated with traditional pollination methods. Various comparative experiments on different pollination methods for strawberry fruit production have been conducted. The development of achenes was the most effective in the order of self-pollination, wind, and insect pollinators [14]. There was no significant difference in fruit fresh weight among self-pollination, pollination via vibration, and hand pollination [59]. In the experiment of ultrasonic radiation on strawberry pollination, 40 and 50 Hz were effective in producing marketable fruits, but it was not easy to establish a correlation between frequency, irradiation time, and the proportion of marketable fruits [15]. These results suggest that external stimulation can enhance pollination efficiency, excluding the effects of insect pollinators. However, existing pollination methods seem to result in a high incidence of malformed strawberries and have not yet entirely replaced the need for insect pollinators.

Herein, strategies to enhance the effectiveness of mechanical pollination were employed by adjusting the VPD conditions and using precise vibrations. The sampled flowers were used to assess the pollination efficiency, and vibrations were applied after exposure to VPD conditions. Various factors must be considered when applying VPD control and vibrations in actual strawberry cultivation. For flowers attached to plants, the response time of anthers varies depending on whether the flowers are attached or detached, influenced by environmental conditions, while the direction of applied vibrations also differs. In various crops, the response tendencies of anther dehiscence were similar between detached and attached flowers; however, differences in response speed were observed [20, 22].

Future studies should explore the outcomes of using vibration pollination in conjunction with insect pollinators and evaluate the feasibility of using solitary vibration pollination. The findings of this foundational research can be utilized as preliminary data before implementing an effective mechanical pollination system. Developing precise control systems for mechanical pollination systems could further be an important solution for enhancing pollination in controlled agricultural environments.

## Conclusions

Based on our findings, this study demonstrates that environmental control strategies, particularly VPD conditions and specific mechanical vibration characteristics, can significantly enhance the self-pollination efficiency of strawberry flowers in indoor vertical farming systems. The results indicate that anther dehiscence and self-pollination are effectively influenced by higher VPD of 2.06 kPa and frequencies of 100 Hz combined with G_rms_ of 30 m s^−2^. These insights offer a novel approach to optimizing pollination processes in indoor vertical farms by reducing reliance on insect pollinators and labor-intensive hand pollination. Implementing such methods can enhance pollination efficiency and support sustainable production in controlled environment agriculture. Future research should investigate the combined effects of vibration pollination and insect pollinators and the feasibility of solitary vibration pollination. This study offers essential data for developing precise and effective mechanical pollination systems, contributing to advancing high-quality fruit production in indoor vertical farming systems.

## Data Availability

No datasets were generated or analysed during the current study.
